# LOSING PERSONHOOD: EXPERIENCES OF INDIVIDUALS CARING FOR A FAMILY MEMBER WITH DEMENTIA

**DOI:** 10.1093/geroni/igad104.0339

**Published:** 2023-12-21

**Authors:** Rachel Carter, Sally Norton, Marian Moskow, Melanie Bobry, Kimberly Van Orden, Kathi Heffner, Marsha Wittink

**Affiliations:** University of Rochester, Rochester, New York, United States; University of Rochester, Rochester, New York, United States; University of Rochester School of Nursing, Rochester, New York, United States; University of Rochester, Rochester, New York, United States; University of Rochester Medical Center, Rochester, New York, United States; University of Rochester Medical Center, Rochester, New York, United States; University of Rochester, Rochester, New York, United States

## Abstract

Over 11 million people in the United States are providing informal care for a person with dementia (PwD). When providing care, family caregivers for a PwD may find their own sense of personhood becomes lost, impacting their social connectedness. This qualitative study characterizes family caregivers’ social connection experiences, goals, barriers, and facilitators. We used a criterion-based purposeful sampling strategy to identify 30 family caregivers for PwD ages 50+ who were lonely (n=20) and not lonely (n=10) according to their UCLA loneliness scale short form scores (not lonely = < 5 and lonely = ≥ 6). We recruited selected participants through the Healthy Aging Research Program (HARP) population study. We then conducted individual, semi-structured interviews with each participant and performed a content analysis on all transcribed interviews, utilizing preset codes based on a theoretical frame and developing novel codes until key themes were identified. Participants reported connections between social connectedness—loneliness, disconnection, and feelings of isolation—and the loss or diminishment of their own personhood. When asked about their past and present social activities, the ones caregivers identified as important allowed them to feel connected to others and to themselves, offering either or both: 1) the opportunity to relate to others in ways they could not relate to the PwD; 2) social and emotional engagement that reinforced their sense of personhood. When social activities did not offer either of these, caregivers experienced disconnection. Results indicate potential ways to intervene to mitigate loss of personhood and promote social connectedness in family caregivers.

